# Pan‐cancer analysis of mutations in open chromatin regions and their possible association with cancer pathogenesis

**DOI:** 10.1002/cam4.4749

**Published:** 2022-04-13

**Authors:** Chie Kikutake, Mikita Suyama

**Affiliations:** ^1^ Medical Institute of Bioregulation Kyushu University Fukuoka Japan

**Keywords:** bioinformatics, cancer genetics, genomics, medical genetics, TCGA

## Abstract

**Background:**

Open chromatin is associated with gene transcription. Previous studies have shown that the density of mutations in open chromatin regions is lower than that in flanking regions because of the higher accessibility of DNA repair machinery. However, in several cancer types, open chromatin regions show an increased local density of mutations in activated regulatory regions. Although the mutation distribution within open chromatin regions in cancer cells has been investigated, only few studies have focused on their functional implications in cancer. To reveal the impact of highly mutated open chromatin regions on cancer, we investigated the association between mutations in open chromatin regions and their possible functions.

**Methods:**

Whole‐genome sequencing data of 18 cancer types were downloaded from the PanCancer Analysis of Whole Genomes and Catalog of Somatic Mutations in Cancer. We quantified the mutations located in open chromatin regions defined by The Cancer Genome Atlas and classified open chromatin regions into three categories based on the number of mutations. Then, we investigated the chromatin state, amplification, and possible target genes of the open chromatin regions with a high number of mutations. We also analyzed the association between the number of mutations in open chromatin regions and patient prognosis.

**Results:**

In some cancer types, the proportion of promoter or enhancer chromatin state in open chromatin regions with a high number of mutations was significantly higher than that in the regions with a low number of mutations. The possible target genes of open chromatin regions with a high number of mutations were more strongly associated with cancer than those of other open chromatin regions. Moreover, a high number of mutations in open chromatin regions was significantly associated with a poor prognosis in some cancer types.

**Conclusions:**

These results suggest that highly mutated open chromatin regions play an important role in cancer pathogenesis and can be effectively used to predict patient prognosis.

## Graphical abstract

In this study, to reveal the impact of highly mutated open chromatin regions on cancer, we analyzed whole‐genome sequencing data of cancer genome. Specifically, we investigated the association between mutations in open chromatin regions and their possible functions.

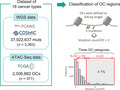

## 1 BACKGROUND

Open chromatin is a locally accessible region of the genome. DNA is usually wrapped around histone proteins to form nucleosomes, and multiple nucleosomes are folded into higher‐ordered chromatin structures. In the heterochromatin (closed chromatin) state, heterochromatin‐associated proteins and silencing factors bind to DNA,^1^ resulting in gene silencing. On the other hand, the euchromatin (open chromatin), a less condensed chromatin, is associated with gene transcription and is found in transcriptionally active gene loci or regulatory regions, such as promoters and enhancers.

Previous studies have shown that fewer mutations occur in open chromatin regions than in the flanking regions.^2^, ^3^ In other words, the mutation density is lower in transcriptionally active regions. It is believed that this mutation distribution in the genome is due to the higher accessibility of open chromatin by the DNA repair system. Indeed, the high chromatin accessibility allows enhanced nucleotide excision repair (NER) and base excision repair activities, leading to a relative decrease in the mutation density in open chromatin regions.^4^ A previous work has demonstrated that, in normal human skin fibroblasts, mutations in transcriptionally active regions and open chromatin regions were repaired more quickly than those in other genomic regions, whereas the regions with the delayed repair were associated with a higher level of cancer‐linked mutations.^4^

The number of mutations is, however, not always uniformly lower within open chromatin regions. Chromatin accessibility is a factor that leads to mutation accumulation in several types of cancer, and some other factors are also associated with mutation accumulation.^5^ A higher mutation density is found in localized regions within DNase I‐hypersensitive sites (DHSs). For example, in melanoma, lung cancer, and ovarian cancer, mutations are enriched in the center of DHSs in gene promoter regions, which are strongly associated with transcription initiation activity.^6^ Similarly, in ubiquitous enhancers (enhancers that strongly promote transcription of enhancer RNAs across all cell lines studied),^7^ the mutation density is significantly higher than that in the flanking regions, and the mutation density ratio between DHS and DHS flanking regions is significantly higher than that in permissive enhancers (enhancers that show bidirectional transcription in a cell‐type‐specific manner) in melanoma, lung cancer, and ovarian cancer.^6^, ^7^ Moreover, the analysis of the distribution of somatic mutations in TF binding sites (TFBSs) in melanoma revealed that active TFBSs, which overlap with open chromatin regions, have a higher mutation rate than flanking regions because of the increased level of UV‐induced damage.^8^, ^9^ The enrichment of mutations in CTCF‐binding sites was observed in some cancer types.^10^, ^11^ This is partially due to DNA‐binding TFs interfering with the NER machinery, resulting in increased DNA mutation rates at the TFBSs. In addition, we previously showed that the number of mutations in open chromatin regions with at least one recurrent mutation is significantly higher than that in open chromatin regions without recurrent mutations.^12^ Thus, the distribution of mutations in open chromatin regions is heterogeneous, with some open chromatin regions being highly mutated.

Although several studies have investigated the mutation distribution in the open chromatin regions of cancer cells or analyzed specific cancer‐related mutations in regulatory regions,^13^, ^14^, ^15^, ^16^ only a few studies have focused on their functional implication of mutation‐enriched open chromatin regions in various cancers. Recently, The Cancer Genome Atlas (TCGA) provided pan‐cancer ATAC‐Seq data^17^ and the PanCancer Analysis of Whole Genomes (PCAWG) project provided 2658 whole‐genome sequencing (WGS) data from 38 cancer types, enabling comprehensive mutation analysis of the entire cancer genome.^18^ In addition, databases such as The Encyclopedia of DNA Elements (ENCODE)^19^ and Roadmap^20^ have made it possible to use epigenomic and higher‐order chromatin structure data generated from various tissues to interpret the functions of certain genomic regions. The accumulation of these data facilitates the comprehensive analysis of mutation‐enriched open chromatin regions and their association with cancer pathogenesis.

In the present study, we analyzed the WGS data from PCAWG^18^ and Catalog of Somatic Mutations in Cancer (COSMIC).^21^ We examined the characteristics and functions of open chromatin regions with mutations. We found an association between the number of mutations in open chromatin regions and the proportion of regulatory regions and genome amplification in several cancer types. Gene ontology (GO) enrichment analysis showed that possible target genes of open chromatin regions with a higher number of mutations were more strongly associated with cancer pathogenesis than those of other open chromatin regions. Finally, we performed a survival analysis using the data on highly mutated open chromatin regions. The results showed a significantly poor prognosis for patients with a high number of mutations in the mutation‐enriched open chromatin regions in some cancer types. Our findings suggest that highly mutated open chromatins can function as regulatory elements in various cancers and might be used as prognostic markers.

## 2 MATERIALS AND METHODS

### 2.1 Datasets

WGS data, copy number variants, and clinical data were downloaded from the PCAWG project.^18^ We also downloaded mutation data of whole‐genome sequenced samples from the COSMIC data repository (CosmicGenomeScreensMutantExport.tsv.gz and CosmicNCV.tsv.gz) (GRCh37, release v92; 27 August 2020).^22^ We focused on 18 solid cancer types: bladder urothelial carcinoma (BLCA), breast adenocarcinoma (BRCA), cervical squamous cell carcinoma and endocervical adenocarcinoma (CESC), colon adenocarcinoma (COAD), esophageal carcinoma (ESCA), glioblastoma multiforme (GBM), head and neck squamous cell carcinoma (HNSC), kidney renal clear cell carcinoma (KIRC), kidney renal papillary cell carcinoma (KIRP), lower‐grade glioma (LGG), liver hepatocellular carcinoma (LIHC), lung adenocarcinoma (LUAD), lung squamous cell carcinoma (LUSC), prostate adenocarcinoma (PRAD), skin cutaneous melanoma (SKCM), stomach adenocarcinoma (STAD), thyroid carcinoma (THCA), and uterine corpus endometrial carcinoma (UCEC) (Table 1). We extracted information regarding mutations in these 18 cancer types from the PCAWG and COSMIC datasets. Single nucleotide polymorphisms with an allele frequency of ≥0.01 assessed by gnomAD (version 2.1.1)^23^ and mutations in samples from TCGA^24^ or the International Cancer Genome Consortium^25^ that were included in the PCAWG data repository were removed from the mutations listed in the COSMIC dataset. Then, we extracted the mutations that were experimentally confirmed by sequencing both cancer and a matched normal tissue or blood from the same patient. Finally, mutations from the PCAWG and COSMIC datasets were merged into a unique gene list for each cancer type and mutations with mappability scores not equal to 1 were filtered out. The human reference genome GRCh37 was used in this study.

**TABLE 1:** Cancer sample size for each dataset

Cancer types	WGS from PCAWG	WGS from COSMIC	ATAC‐Seq from TCGA
BLCA	23	9	10
BRCA	214	71	75
CESC	20	0	4
COAD	60	31	41
ESCA	98	107	18
GBM	41	182	9
HNSC	44	0	9
KIRC	111	7	16
KIRP	33	4	34
LGG	18	182	13
LIHC	349	362	17
LUAD	38	175	22
LUSC	48	148	16
PRAD	286	12	26
SKCM	107	301	13
STAD	75	95	21
THCA	48	13	14
UCEC	51	0	13
Total	1664	1699	371

### 2.2 Classification of open chromatin regions according to the number of mutations

To define open chromatin regions, ATAC‐Seq data of the selected 18 cancer types were downloaded from TCGA.^17^ The ATAC‐Seq data were converted from human genome assembly GRCh38 to GRCh37 using the liftOver program.^26^ In the ATAC‐Seq data obtained from TCGA, peak location is defined as a 500 bp region. We used the 500 bp regions (=250 bp on either side of the midpoint) for open chromatin analysis in this study. The midpoint of the converted open chromatin regions was calculated, and the 250 bp region on either side of the midpoint was defined as an open chromatin region. The number of mutations located in open chromatin regions was counted in each cancer type using the mutation dataset obtained as described above.

Open chromatin regions were divided into three categories according to the number of mutations: (1) open chromatin regions without mutation (N‐OC), (2) the top 1% of open chromatin regions with the highest number of mutations (H‐OC), and (3) other open chromatin regions (L‐OC) (Table 2 and Table S1). Since the numbers of mutations are discrete values, we set the value not exceeding 1% as the cutoff for H‐OC.

**TABLE 2:** Number of mutations in the three categories of open chromatin

Cancer types	Open chromatin category		
	N	L	H
CESC, GBM, HNSC, KIRP, LGG, PRAD, THCA	0	1	≥2
BLCA, BRCA, ESCA, KIRC, LUAD, LUSC, STAD, UCEC	0	1–2	≥3
LIHC	0	1–3	≥4
COAD	0	1–4	≥5
SKCM	0	1–7	≥8

### 2.3 Evaluation of the open chromatin state

We analyzed the epigenetic dataset available for 23 tissues derived from 15 cancer types with the core 15‐state model calculated by ChromHMM^27^ in the Roadmap Epigenomics Project^20^, ^28^ (Table S2). The proportion of each 15‐state to the total open chromatin length was calculated for the three open chromatin categories. The proportion of each 15‐state in the entire genome was calculated as a control.

### 2.4 Evaluation of TF footprinting in open chromatin regions

TF footprint data (false‐positive rates <0.05) were used for the analysis of TF binding to open chromatin regions.^29^ Specifically, we used datasets from 30 cancer cell lines or primary cells derived from 12 cancer types (Table S3). The TF footprint length per open chromatin region for the three categories was calculated for each cancer type.

### 2.5 Investigation of genome amplification in open chromatin regions

Data regarding the copy number in each sample from the PCAWG repository were used to obtain the copy number for each open chromatin region. The average copy number in each open chromatin region was calculated for each cancer type. Regions with an average copy number of ≥4 were defined as amplified open chromatin regions.^30^ The proportion of amplified open chromatin regions was calculated for each cancer type. The same analysis was performed for regions outside the open chromatin regions.

To investigate the co‐occurrence of mutations and genome amplification in each open chromatin region, samples were divided into four groups: (1) regions with both mutations and genome amplification, (2) regions with only mutations, (3) regions with only genome amplification, and (4) regions with neither mutation nor genome amplification. Then, we calculated, for each cancer type and open chromatin region, the observed and expected number of samples with mutations and genome amplification in the open chromatin region and analyzed the ratio using the *t*‐test.

### 2.6 Extracting possible target genes of open chromatin regions

To identify the genes from which the promoter overlaps with open chromatin regions, we defined a promoter region as ±3000 bp around the transcription start site (TSS) using GTF files (Homo_sapiens.GRCh37.87.gtf) downloaded from Ensembl. To identify genes spatially in contact with open chromatin regions, RNA Polymerase II ChIA‐PET data from the MCF7 human breast cancer cell line were downloaded from ENCODE.^19^ These two gene lists were merged resulting in a list of possible target genes of open chromatin regions. GO analyses were performed using Metascape 3.5^31^ and R package clusterProfiler (version 3.14.3). We used Metascape 3.5 and R package disgenet2r (version 0.1.1) to analyze the association between genes and human diseases.^32^

### 2.7 Survival analysis according to the number of mutations in open chromatin regions

Cox proportional hazards model in R survival package (version 3.2‐11) was used to estimate hazard ratios (HRs) and their 95% confidence intervals. We used thresholds for the number of mutations in open chromatin regions ranging from the 10th to 90th percentiles and defined the value that distinguished samples with the highest statistical significance as the final threshold.^33^

To analyze all samples, regardless of the cancer type, we used the following variables as covariates: cancer type, tumor mutation burden (TMB), total number of amplified open chromatin regions, gender, and age at diagnosis. The same covariates excluding the cancer type were used for the analysis of samples from each cancer type. We also used cancer stage data if available. The TMB and total number of amplified open chromatin regions were binarized based on the median values and the cancer stage was divided into two categories (stages I–II vs. stages III–IV). Using 100 times 5‐fold cross‐validation, Harrell's concordance index (C‐index)^34^ was calculated to assess the discriminative power of the prognostic models.

### 2.8 Statistical analyses

Statistical analyses were performed using the R software version 4.0.1 (R Project for Statistical Computing). Jonckheere–Terpstra trend test and Cochran–Armitage trend test were used to determine if there was a significant trend among the three categories. Benjamini–Hochberg procedure was used to adjust for multiple testing.^35^ Statistical analyses were two‐sided and a *p*‐value of <0.05 was considered statistically significant (*p < 0.05, **p < 0.01, and ***p < 0.001).

## 3 RESULTS

### 3.1 Classification and genomic characteristics of open chromatin regions

**FIGURE 1:**
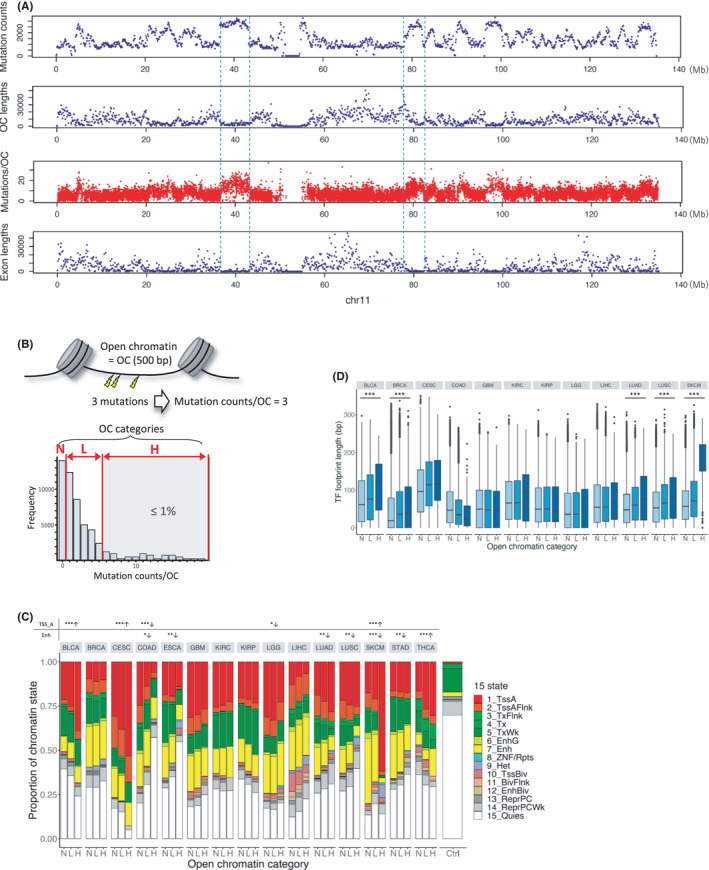
Mutation distribution and characteristics of open chromatin regions. (A) Distribution of the total number of mutations (top), total length of open chromatin regions (upper), total number of mutations per open chromatin region (lower), and total exon lengths (bottom) per 100 kb on chromosome 11. “OC” on the horizontal axis represents mutations within open chromatin regions. (B) Open chromatin categories used in this study. Open chromatin regions were divided into three categories defined as N‐OC, L‐OC, and H‐OC based on the number of mutations. (C) Proportion of the frequency of 15 chromatin states in open chromatin regions for 15 cancer types. The horizontal axis represents the three categories of open chromatin regions in each cancer type and the vertical axis represents the proportion of 15 chromatin states. Ctrl represents the average proportion of the 15 chromatin states in the whole genome. Asterisks represent the cancer types with a significantly higher proportion of the chromatin state “1_TssA” or “7_Enh” in H‐OC regions. The upward arrows indicate that the proportion is significantly higher for open chromatins with more mutations, whereas the downward arrows indicate that the proportion is significantly lower for open chromatins with more mutations. (D) TF footprint length per open chromatin in the three categories for 12 cancer types. The horizontal axis represents the three categories of open chromatin for 12 cancer types and the vertical axis represents the TF footprint length per open chromatin (bp)

To analyze the number of mutations in open chromatin regions from cancer samples, we used the WGS data from PCAWG. Out of 2658 samples of 38 cancer types in PCAWG, 1664 samples of 18 cancer types with ATAC‐Seq data were used in the present analysis (Table 1). We also used a mutation dataset in COSMIC derived from WGS comprising 1699 samples from 18 cancer types (Table 1). These mutation datasets were merged into a unique mutation list for each cancer type. The total number of unique mutations was 37,922,637. ATAC‐Seq data from 371 samples from the selected 18 cancer types were downloaded from TCGA. The cancer type with a maximum number of open chromatin regions (214,911) was BRCA and that with a minimum number (55,895) was CESC. The distribution of unique mutations per 100 kb bin across the entire genome showed that there was a general trend of mutation enrichment in the regions with a higher density of open chromatin regions and a relatively low density of exons (Figure 1A). Focusing on the number of mutations per open chromatin region revealed that the number of mutations in each open chromatin region was variable.

We counted the number of unique mutations in each open chromatin region for each cancer type from the ATAC‐Seq data. To investigate the relationship between the number of mutations in open chromatin regions and cancer pathogenesis, we first classified open chromatin regions into three categories according to the number of mutations for each cancer type. The categories N, L, and H contained 89,744 ± 27,588, 21,191 ± 18,758, and 558 ± 446 (average ± standard deviation) open chromatin regions, respectively (hereafter, the N, L, and H open chromatin regions are named N‐OC, L‐OC, and H‐OC, respectively.) (Figure 1B, Tables S1 and S2).

First, we investigated the mutation spectrum in open chromatin regions. For this, we used regions without open chromatins as the control and compared the difference in the mutation spectrum between inside and outside open chromatin regions. We found that the proportions of C>G and C>T mutations in open chromatin regions were significantly higher than those outside open chromatin regions in all cancer types analyzed except KIRP, LGG, PRAD, and SKCM (Figure S1A). The APOBEC signature is based on C>T and C>G mutations associated with various cancer types.^36^, ^37^ Therefore, the difference in the proportions of C>T and C>G mutations between open and closed chromatin regions might be partly attributed to the APOBEC enzyme activity, through which mutations are enriched in regions with high chromatin accessibility.^38^ The comparison of L‐OC and H‐OC mutation spectra revealed that, in SKCM, the proportion of C>T mutation in H‐OC regions was higher than that in L‐OC regions (Fisher's exact test: adjusted *p* = 2.06e‐22) (Figure S1B). This finding was consistent with that of a previous work showing an increased number of UV‐induced C>T mutations in promoter regions in melanoma.^9^

Next, to assess the open chromatin state, we used the chromatin state data from Roadmap defined by ChromHMM. The chromatin state data of the 15 cancer types analyzed were found in Roadmap with the exception of those for HNSC, PRAD, and UCEC (Table S3). We calculated the proportion of each chromatin state in each of the three open chromatin categories. For control, we calculated the proportion of each chromatin state in the whole genome. The results showed that the proportion of active TSS state increased with the number of mutations within open chromatin regions in BLCA, CESC, and SKCM (Cochran–Armitage trend test: adjusted *p* = 1.86e‐9, 2.66e‐12, and 4.01e‐48, respectively) (Figure 1C). In addition, the proportion of enhancer state increased with the number of mutations within the open chromatin regions in THCA (Cochran–Armitage trend test: adjusted *p* = 8.65e‐7). We also performed the same analysis with different definitions of H‐OC: the top 2% and 5% of open chromatin regions with the highest number of mutations. These results were similar to those obtained when H‐OC was defined based on the 1% (Figure S2). This is because the threshold of mutation counts did not change in most cancer types, even if different percentages (1%, 2%, or 5%) were used as the cutoff value (Tables S4 and S5). To validate the results of the chromatin state, we compared the proportions of open chromatin regions with a certain histone modification to that of all open chromatin regions for each histone modification using ChIP‐Seq data of H3K4me1, H3K4me3, and H3K27ac from the Roadmap Epigenomics Project (excluding BLCA, KIRC, and KIRP of H3K27ac owing to the lack of the data) (Figure S3). The results supported those obtained using ChromHMM; for example, SKCM showed a decrease in the number of H3K4me1 peaks (=enhancer state) in H‐OC regions. These results indicate that open chromatin regions with a high number of mutations in at least four cancer types are more likely to function as regulatory regions. The ChromHMM data for SKCM were derived from normal cells. Therefore, the decrease in the proportion of enhancers in the H‐OC region in SKCM is not a cancer‐specific phenomenon.

Then, to investigate the function of H‐OC as a regulatory element, we measured the TF footprint length within each open chromatin region. We focused only on open chromatin regions annotated as active TSS or enhancer by ChromHMM. Footprinting data^29^ were retrieved for 13 cancer types, namely BLCA, BRCA, CESC, COAD, GBM, KIRC, KIRP, LGG, LIHC, LUAD, LUSC, PRAD, and SKCM (Table S6). The TF footprint length was significantly longer in open chromatin regions with more mutations than in those with less mutations in BLCA, BRCA, LUAD, LUSC, and SKCM (Jonckheere–Terpstra trend test: all adjusted *p* < 2.2e‐16) (Figure 1D). TF binding to regulatory elements in these five cancer types might inhibit the repair of mutations in regulatory regions, leading to more mutations in open chromatin regions.^6^ These results support the findings of a previous study showing a significant increase in the mutation density in promoter DHS compared with that in flanking regions in melanoma and lung cancer.^6^

### 3.2 Genome amplification of open chromatin regions

To evaluate whether N‐OC, L‐OC, and H‐OC regions are possibly carried on extrachromosomal DNA (ecDNA) in cancer cells, we examined the copy number of open chromatin regions and the mutations therein. The chromatin is highly accessible in ecDNA^39^, ^40^ and ecDNA can be detected by ATAC‐Seq because the nucleosome structure is less compact.^41^ Often, ecDNA carries amplified oncogenes and regulatory regions controlling gene expression levels and is associated with cancer pathogenesis and therapeutic resistance.^30^, ^42^

**FIGURE 2:**
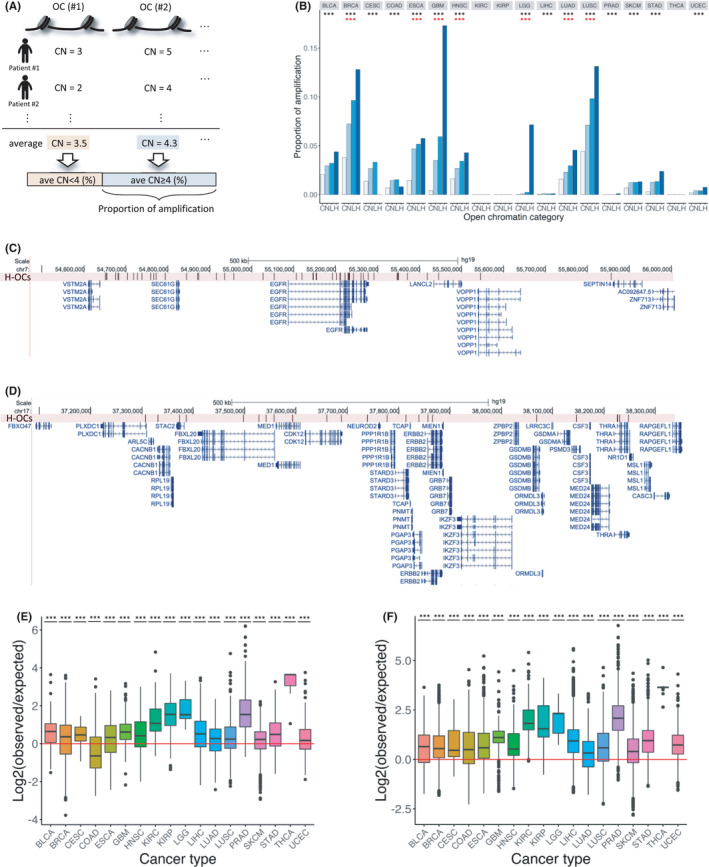
Proportion of genome amplification in open chromatin regions. (A) Outline of the analysis for genome amplification in open chromatin regions. The average copy number (ave CN) was determined for each open chromatin region and the proportion of amplified open chromatin regions was calculated. (B) Proportion of open chromatin regions with an average copy number of ≥4 in 18 cancer types. The horizontal axis represents regions outside open chromatin regions (C: Control) and the three categories of open chromatin regions (N, L, and H) for each cancer type. The black asterisks represent the cancer types with a significantly higher proportion of genome amplification in open chromatin regions than that outside the open chromatin regions. The red asterisk represents the cancer types with a significantly higher proportion of genome amplification in H‐OC regions than those in other open chromatin categories. (C) Genomic region around *EGFR* with H‐OC regions accumulation in GBM. The black vertical lines represent the positions of H‐OC region. (D) Genomic region around *ERBB2* with the accumulation of H‐OC regions in BRCA. (E) Ratio between the observed sample numbers with the co‐occurrence of mutation and genome amplification in each H‐OC region and the expected sample numbers. Red line: ratio = 1. (F) Ratio between the observed sample numbers with the co‐occurrence of mutation and genome amplification in each L‐OC region and the expected sample numbers. Red line: ratio = 1

The average copy number of each open chromatin region was calculated in each cancer type using the copy number of the open chromatin region from each sample. Next, the number of open chromatin regions with an average copy number of ≥4 was determined. This cutoff was adopted because it has been used as a characteristic of ecDNA.^30^ Finally, the proportion of the open chromatin regions with an average copy number of ≥4 was evaluated in each cancer type and was defined as the proportion of amplification (Figure 2A). To compare the proportion of amplification in open chromatin regions with that in the control region, we used regions outside the open chromatin regions as control (Figure 2B). We found that for 15 cancer types, the proportion of amplification was significantly higher in open chromatin regions than that outside open chromatin regions (chi‐square test: adjusted *p* = 2.30e‐84 for BLCA, *p* < 2.2e‐16 for BRCA, *p* = 1.24e‐115 for CESC, *p* = 4.08e‐179 for COAD, *p* < 2.2e‐16 for ESCA, *p* < 2.2e‐16 for GBM, *p* = 1.10e‐129 for HNSC, *p* = 5.02e‐23 for LGG, *p* = 4.83e‐13 for LIHC, *p* = 2.93e‐108 for LUAD, *p* < 2.2e‐16 for LUSC, *p* = 5.59e‐3 for PRAD, *p* = 2.39e‐86 for SKCM, *p* < 2.2e‐16 for STAD, and *p* = 1.26e‐40 for UCEC). We then compared the proportion of amplification in the three categories of open chromatin regions and found that the proportion of amplified open chromatin regions increased with the number of mutations in open chromatin regions in seven cancer types (Cochran–Armitage trend test: adjusted *p* = 6.65e‐77, *p* = 2.92e‐3, *p* = 1.88e‐47, *p* = 8.38e‐6, *p* = 5.28e‐4, *p* = 5.85e‐9, and *p* = 5.88e‐50 for BRCA, ESCA, GBM, HNSC, LGG, LUAD, and LUSC, respectively) (Figure 2B). These results support previous findings showing that ecDNA is present in these cancer types.^30^, ^43^ We also performed a detailed analysis of the genomic position of the highly amplified (copy number ≥4) H‐OC regions in GBM (17.3%; the highest value) and BRCA (12.8%; the second‐highest value). We found that most H‐OC regions (79.7%) with genome amplification in GBM were located within 500 kb from the TSS of epidermal growth factor receptor (*EGFR*) (Figure 2C). A recent study already demonstrated that ecDNA with focally amplified *EGFR* tends to contain mutations.^44^ In BRCA, 10.1% of H‐OC regions with genome amplification were located within 500 kb from TSS of the receptor tyrosine‐protein kinase erbB‐2 (*ERBB2*) (Figure 2D). It has been reported that *ERBB2* is likely to be amplified in breast cancer.^45^ These results suggest that some regulatory regions around oncogenes are amplified as ecDNA in several cancers.

To evaluate whether amplification and mutations occur independently in open chromatin regions, we created a 2 × 2 contingency table for each open chromatin region using the number of samples corresponding to two items: the presence/absence of genome amplification (copy number ≥4) and presence/absence of mutations. Then, we performed Fisher's exact test for each cancer type (Table S7 and Figure 2E,F). In most cancer types, the amplified open chromatin regions and the occurrence of mutations were not independent but were either positively correlated or mutually exclusive. To investigate whether mutations were likely to occur in open chromatin regions prone to amplification, we calculated the expected number of samples with co‐occurrence and compared them with the observed values (Table S7 and Figure 2E,F). The statistical analysis showed that the ratios of all observed to expected values, except the ratio for H‐OC regions in COAD (*t*‐test: adjusted *p* = 0.470), were above 1, which indicated a positive relationship. These results suggest that, in most cancer types, mutations and genomic amplification tend to co‐occur in open chromatin regions.

### 3.3 Investigation of possible target genes of open chromatin

**FIGURE 3:**
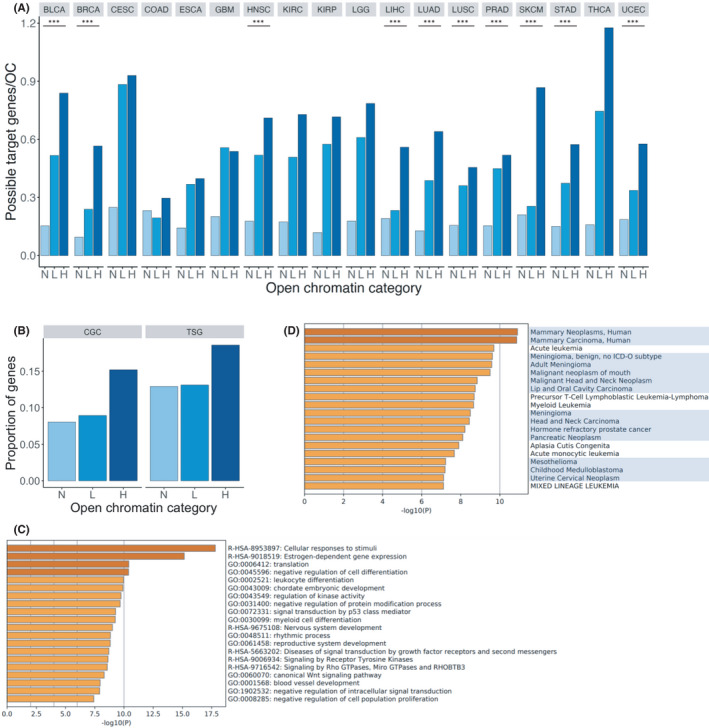
Characteristics of possible target genes of H‐OC regions. (A) Number of possible target genes for each open chromatin region category. The horizontal axis represents the three categories of open chromatin regions (N, L, and H) in 18 cancer types. The black asterisks represent the cancer types with a significantly higher number of possible target genes per open chromatin in H‐OC regions than those in other open chromatin categories. (B) Proportion of genes present in the Cancer Gene Census (CGC) and tumor suppressor genes (TSGs) registered in TSGene 2.0 for each of the three categories. The horizontal axis represents the three categories of open chromatin regions (N, L, and H), and the vertical axis represents the proportion of cancer‐related genes among the selected 1007 genes. (C) GO analysis using the possible target genes of H‐OC regions. The horizontal axis represents the −log10(*p*‐value), and the vertical axis represents the GO terms arranged in the order of descending *p*‐values. (D) The relationship between the possible target genes of H‐OC regions and human diseases was obtained using the DisGeNET database. The horizontal axis represents the −log10(*p*‐value), and the vertical axis represents the human diseases arranged in the order of descending *p*‐values

To identify the genes transcriptionally regulated by open chromatin, we extracted genes with promoters either overlapping or in spatial contact with open chromatin regions from the ChIA‐PET dataset. Because only a limited amount of public ChIA‐PET data was available, we applied to all cancer types the ChIA‐PET data derived from the MCF7 breast cancer cell line, which has the highest number of interactions between two different genomic regions. In this analysis, we focused only on the protein‐coding genes and defined promoter as the region extending 3000 bp upstream and 3000 bp downstream of the gene's TSS. The results showed that in 10 cancer types, namely BLCA, BRCA, HNSC, LIHC, LUAD, LUSC, PRAD, SKCM, STAD, and UCEC, the number of possible target genes per H‐OC region was significantly higher than those per L‐OC and N‐OC regions (Cochran–Armitage trend test: adjusted *p* = 1.13e‐131 for LIHC, *p* = 3.19e‐153 for SKCM, and *p* < 2.2e‐16 for the eight other cancer types) (Figure 3A). The absence of significance for eight cancer types (CESC, COAD, ESCA, GBM, KIRC, KIRP, LGG, and THCA) might be due to the limited number of open chromatin regions or possible target genes. We found 4352 possible target genes of H‐OC regions, 16,966 for L‐OC regions, and 18,227 for N‐OC regions. These gene lists were generated by counting unique genes in the 18 cancer types. Among these, 1007 (H‐OC), 15,600 (L‐OC), and 17,477 (N‐OC) genes were present in multiple cancer types and hence were expected to be more associated with cancer than the other genes in relevant regions. We used these shared genes in the subsequent analysis.

To examine the characteristics of the shared genes in each category of open chromatin, we compared the proportion of cancer‐related genes included in the gene lists. Since a substantial number of genes were included in the gene lists for L‐OC and N‐OC regions, we randomly selected 1007 genes, to match the number of genes in H‐OC regions, from the L and N gene lists 100 times. We calculated the average proportion of genes present in the Cancer Gene Census (CGC) (723 genes)^22^ and tumor suppressor genes (TSGs) registered in TSGene 2.0 (1217 genes) for each of the three categories.^46^ We found that the proportion of genes registered in the CGC was significantly higher among the genes in H‐OC regions (153/1007 = 0.152) than among those in L‐OC regions (average 90/1007 = 0.089) and N‐OC regions (average 81/1007 = 0.080) (Cochran–Armitage trend test: adjusted *p* = 2.16e‐7) (Figure 3B). Moreover, the proportion of TSGs registered in TSGene 2.0 was significantly higher among genes in H‐OC (187/1007 = 0.186) than among those in L‐OC regions (average 132/1007 = 0.131) and N‐OC regions (average 130/1007 = 0.130) (Cochran–Armitage trend test: adjusted *p* = 3.56e‐4) (Figure 3B). These results indicate that cancer‐related genes were likely enriched among the possible target genes of open chromatin regions with more mutations.

To further analyze the characteristics of the shared genes for H‐OC regions, we performed a pathway and process enrichment analysis (Figure 3C). We found that these genes were highly associated with ontology terms related to cellular response to stimuli and kinase activity, as well as terms directly related to cancer or carcinogenesis. To interpret the functions of the shared genes for H‐OC regions, we also analyzed the relationship between these genes and human diseases using the DisGeNET database.^32^ We first obtained data on human diseases associated with genes for H‐OC regions. Then, we extracted data on diseases with adjusted *p* < 0.01 and counted the number of cancer‐related diseases. To compare the data of cancer‐related genes with those of control genes, we performed the same analysis for 1007 genes randomly selected from the gene lists for L‐OC and N‐OC regions 100 times. We identified 39 cancer‐related diseases (147 diseases were extracted) using the shared genes in H‐OC regions, an average of 0.03 cancer‐related diseases (in average 0.20 diseases were extracted) using the shared genes for L‐OC regions, and 0 cancer‐related diseases (average 0.12 diseases were extracted) using the shared genes for N‐OC regions (Figure 3D). The proportion of the number of cancer‐related diseases to the number of extracted diseases using the genes for H‐OC regions was significantly higher than that for L‐OC (*t*‐test: adjusted *p* = 2.2e‐16) and N‐OC regions (*t*‐test: adjusted *p* = 2.2e‐16). Based on these results, it is likely that open chromatin regions with a high number of mutations are strongly associated with the regulation of cancer‐related genes.

### 3.4 Relationship between mutations in open chromatin regions and patient survival

**FIGURE 4:**
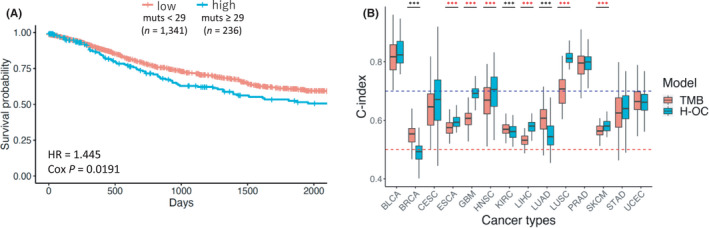
Survival analysis according to the number of mutations in H‐OC regions. (A) Kaplan–Meier curves of samples with <29 (red line) or ≥29 (green line) mutations in H‐OC regions. The horizontal axis represents the time (in days), and the vertical axis represents the survival probability. (B) Comparison of the C‐indexes obtained with the Cox regression model using the TMB (red box) and those obtained using the number of mutations in H‐OC regions (green box) in 14 cancer types. The black asterisk represents the cancer types with a significantly higher C‐index obtained using the TMB model. The red asterisk represents the cancer types with a significantly higher C‐index obtained using the H‐OC model. The C‐index was calculated by 100 times 5‐fold cross‐validation. The red line represents a C‐index of 0.5, and the blue line represents a C‐index of 0.7

Overall, our findings suggest that the mutations in H‐OC regions may have an impact on cancer pathogenesis. To further evaluate the effect of these mutations on cancer pathogenesis, we examined whether the mutations in H‐OC regions affected the prognosis of cancer patients. Using clinical data from PCAWG (*n* = 1577), HRs for the number of mutations in H‐OC regions were estimated by a multivariable Cox proportional hazards model. We used the following variables as covariates: cancer type, TMB, the total number of amplified H‐OC regions, gender, and age at diagnosis. The samples were divided into two groups based on the number of mutations in H‐OC regions: ≥29 mutations (top 15%), *n* = 236, and <29 mutations (bottom 85%), *n* = 1341. The results showed that samples with a higher number of mutations in H‐OC regions were significantly associated with a poorer prognosis than samples with fewer mutations (*p* = 0.0191) (Table 3 and Figure 4A). In contrast, samples with a lower TMB were significantly associated with a poorer prognosis than samples with more mutations (*p* = 5.70e‐3).

**TABLE 3:** Cox regression analysis for all cancer types

Variable	Using the number of mutations in OC^a^	
	Hazard ratio (95% CI)	*p*‐value^b^
The number of mutations in H‐OC		
<29	1.000	–
≥29	1.447 (1.062–1.972)	0.0191*
TMB		
<median	1.000	–
≥median	0.680 (0.517–0.894)	5.70e‐3**
Total number of amplified OC^a^		
<median	1.000	–
≥median	1.058 (0.860–1.302)	0.594
Gender		
Female	1.000	–
Male	1.091 (0.855–1.393)	0.481
Age at diagnosis	1.015 (1.006–1.025)	1.19e‐3**

^a^ OC represents open chromatin regions.

^b^ *p < 0.05 and **p < 0.01.

We also performed a survival analysis for the 18 cancer types. Because of the small sample size, the survival analysis could not be performed for four cancer types (COAD, KIRP, LGG, and THCA). We used the following variables as covariates: cancer type, TMB, the total number of amplified H‐OC regions, gender, age at diagnosis, and stage if the cancer stage data is available (BRCA, ESCA, KIRC, LIHC, PRAD, SKCM, and STAD). The prognosis was significantly poorer for patients with a greater number of mutations within H‐OC regions in four cancer types (adjusted *p* = 0.0497, *p* = 7.55e‐4, *p* = 2.80e‐4, *p* = 0.0391, and *p* = 0.0173 for ESCA, LIHC, LUSC, SKCM, and UCEC, respectively) (Figure S4). On the other hand, a lower number of mutations within H‐OC regions was significantly associated with a poor prognosis for patients with GBM (adjusted *p* = 0.0329) (Figure S4). These results indicate that the number of mutations in H‐OC regions is associated with patient survival in several cancers. To examine the difference in the possible target genes in H‐OC regions between the five cancer types (ESCA, LIHC, LUSC, SKCM, and UCEC) and GBM, which showed a different trend of prognosis, we extracted data on oncogenes and TSGs registered in CGC and TSGene 2.0, respectively, using the gene lists for these six cancer types. We compared the proportion of the number of oncogenes and TSGs included between the two groups of cancer types and found that there were no significant differences between the two analyzed groups of cancer types (oncogene: *p* = 0.621, TSG: *p* = 0.147). These results suggest that patient prognosis is likely to be associated with not only mutations in the regulatory regions of cancer‐related genes but also mutations in other regions.

To compare the effect of the number of mutations in H‐OC regions with the effect of the TMB on prognosis, we evaluated the accuracy of clinical outcome prediction using the C‐index calculated by 100 times 5‐fold cross‐validation. We used the following variables as covariates: total number of amplified H‐OC regions, gender, and age at diagnosis. Data for COAD, KIRP, LGG, and THCA were not analyzed because of the small sample sizes. However, among the 14 cancer types analyzed, the C‐indices for six types (ESCA, GBM, HNSC, LIHC, LUSC, and SKCM) were significantly higher in the model using the number of mutations in H‐OC regions than those obtained with the TMB‐based model (*t*‐test: adjusted *p* = 8.97e‐9, 6.83e‐49, 5.72e‐4, 5.80e‐37, 2.07e‐43, and 1.67e‐8 for ESCA, GBM, HNSC, LIHC, LUSC, and SKCM, respectively) (Figure 4B). On the other hand, for BRCA, KIRC, and LUAD, the C‐indices were significantly higher in the model that used the TMB than those obtained with the model using the number of mutations in H‐OC regions (*t*‐test: adjusted *p* = 1.46e‐16, 2.40e‐2, and 2.92e11 for BRCA, KIRC, and LUAD, respectively). These results suggest that the number of mutations in H‐OC regions is a factor predicting patient prognosis complementary to the TMB.

To validate the effect of H‐OC mutations on patient prognosis, we performed the same survival analysis as described above using WGS data for LUSC (*n* = 103) derived from The National Cancer Institute's Clinical Proteomic Tumor Analysis Consortium (CPTAC).^47^ First, the number of mutations in H‐OC regions from LUSC samples was counted as defined above. Then, the HR for the number of mutations in H‐OC regions was estimated by a multivariable Cox proportional hazards model using the following variables as covariates: TMB, the total number of amplified H‐OC regions, gender, and age at diagnosis. The results showed a link between the high number of mutations in H‐OC regions and a poor prognosis (Figure S5), confirming our finding in an independent cohort.

## 4 DISCUSSION

Our previous study showed that open chromatin regions with recurrent mutations tend to accumulate mutations other than recurrent mutations.^12^ Because open chromatins include regulatory elements and TFBS mutations can lead to the dysregulation of target cancer‐related genes, we hypothesized that open chromatin regions with a high number of mutations would be associated with cancer. Here, we focused on mutation‐enriched open chromatin regions and explored the functional and clinical implications using the WGS dataset from PCAWG and COSMIC to examine the characteristics of the mutation‐enriched open chromatin regions. In most cancer types (except COAD, KIRC, and KIRP), we found that open chromatin regions with a high number of mutations are more likely to function as regulatory regions on the basis of the combination of analysis involving the chromatin state, genome amplification, and the number of the possible target genes. These findings are supported by the results that the possible target genes of open chromatin regions with a high number of mutations were more likely to be associated with cancer pathogenesis. Moreover, we found that the number of mutations in H‐OC regions is associated with the patient prognosis in six cancer types, some of which have had no effect on prognosis when considering the TMB.

LUSC was one of the cancer types in which more mutations in H‐OC regions were significantly associated with poor prognosis. In LUSC, the proportion of genome amplification in H‐OC regions was higher than that in other open chromatin regions. Moreover, mutations and genome amplification in H‐OC regions co‐occurred in LUSC. The higher amplification ratio in H‐OC regions was considered to be partially due to ecDNA.^30^ In cancer cells, multiple copies of genomic regions exist as ecDNA, in which a higher probability of somatic mutations is observed than that noted in chromosomes. The evolution of ecDNA is independent of chromosomal DNA and is faster than that of regular chromosomes.^48^ In a previous study, extrachromosomal gain‐of‐function mutations were identified as amplification‐linked extrachromosomal mutations in several cancer types, including LUSC.^44^ Moreover, ecDNA amplifications are associated with cancer aggressiveness.^30^ Our results support these previous findings as, in LUSC, the accumulation of mutations in ecDNA may affect patient prognosis.

LIHC samples with more mutations in H‐OC regions were also associated with poor prognosis. Because of the low proportion of amplification in H‐OC regions, we considered that ecDNA was not linked to the accumulation of mutations in the open chromatin regions in LIHC. The number of genes with promoters possibly regulated by H‐OC regions was significantly higher than that of genes with promoters regulated by other open chromatin regions. Moreover, the proportion of enhancer chromatin state in H‐OC regions was relatively higher than that in other cancer types. These results suggest that H‐OC regions are likely to function as regulatory regions, such as enhancers. A recent study showed that mutations in coding and regulatory regions play important roles in carcinogenesis and gene expression.^49^ Recurrently mutated regulatory regions were identified in another work based on a large‐scale whole‐genome analysis in liver cancer.^50^ Therefore, it is likely that some of the mutations in the H‐OC regions in LIHC may be associated with the control of gene expression and affect liver cancer pathogenesis.

TMB is a predictive marker of the response to immune checkpoint inhibitors and is routinely tested in clinical practice. Several studies have linked high TMB with a high response rate of immune checkpoint inhibitors in certain cancer types, such as lung cancer and melanoma.^51^, ^52^ Moreover, TMB was proposed as a prognostic factor in cancer patients. TMB had a significant impact on the prognosis for 14 out of 20 cancer types.^33^ The 20 cancer types were divided into three groups: cancer types with a high TMB and a poor prognosis, those with a low TMB and a poor prognosis, and those with no difference in TMB. Four cancer types in which TMB had no impact on the prognosis were included in the present analysis. For two of them (LUSC and SKCM), the number of mutations in H‐OC regions had a significant impact on the prognosis. These results indicate that TMB and mutations in H‐OC regions may have different effects on cancer and that the number of mutations in H‐OC regions might be a novel prognostic factor complementing TMB. Therefore, it is necessary to consider both TMB and mutations in functionally important open chromatin regions for the analysis of cancer pathogenesis. Although not all H‐OC regions have an impact on prognosis, the combination of mutations within some of the H‐OC regions possibly affects the prognosis. Many studies have been conducted to identify driver mutations in non‐coding regions and only a few single non‐coding mutations affected the expression levels of target genes.^13^, ^15^, ^53^ Therefore, to identify novel non‐coding driver mutations, a combination of mutations in several open chromatin regions should be considered.

This study has some limitations. The ATAC‐Seq data used in this study were derived from a limited number of samples, and more open chromatin regions can be examined if the number of available ATAC‐Seq data increases. A previous study has shown that an increasing number of detected open chromatin regions is expected with an increasing sample size.^17^ Therefore, with sufficient ATAC‐Seq data, novel open chromatin regions important for cancer development may be identified. Another limitation of this study is that the ATAC‐Seq data did not originate from the same individuals as those from whom the WGS data were obtained. Using WGS, ATAC‐Seq, and clinical data from the same individuals and an adequate sample size may facilitate the analysis of mutation distribution in open chromatin regions for each individual,^54^ leading to the understanding of intertumoral heterogeneity.

## 5 CONCLUSIONS

In conclusion, we provide new insights into the characteristics of open chromatin regions and show that mutations in open chromatin regions are a prognostic factor in various cancer. After validating our study findings, the characteristics of open chromatin regions can be used as a prognostic factor in clinical practice. In addition, experimental validation of the effects of individual mutations in open chromatin regions will allow the identification of novel cancer‐associated regulatory sequences or gene regulatory mechanisms. These open chromatin regions may not only provide insights for elucidating the mechanisms of cancer development but also represent novel drug targets.

## CONFLICT OF INTEREST

The authors declare no potential conflicts of interest.

## AUTHOR CONTRIBUTIONS

Chie Kikutake contributed to the conception and design of the study, writing the original draft, and data analysis. Mikita Suyama contributed to data review and interpretation, editing final draft, supervision, and project administration. All authors reviewed and approved the article.

## PATIENT CONSENT

Not applicable.

## PERMISSION TO REPRODUCE MATERIAL FROM OTHER SOURCES

Not applicable.

## CLINICAL TRIAL REGISTRATION

Not applicable.

## ETHICS STATEMENT

Not applicable.

## Supporting information


Figures S1‐S5

Tables S1‐S7

## Data Availability

The results shown here are mainly based on data generated by PCAWG: https://dcc.icgc.org/pcawg/ and COSMIC: https://cancer.sanger.ac.uk/cosmic. ATAC‐Seq data in TCGA were derived from https://gdc.cancer.gov/about‐data/publications/ATACseq‐AWG/. SNP in human genome data was derived from https://gnomad.broadinstitute.org/. The datasets used as annotation for open chromatin regions are available in Roadmap Epigenomics Project: http://www.roadmapepigenomics.org/, ENCODE: https://www.encodeproject.org/, TF footprinting data: https://www.vierstra.org/resources/dgf, Metascape: https://metascape.org/, and UCSC genome browser: http://genome.ucsc.edu/, CPTAC: https://proteomics.cancer.gov/programs/cptac.
