# Supplementary figures and images for: Pan‐cancer analysis of mutations in open chromatin regions and their possible association with cancer pathogenesis
Source: Cancer Med. 2022 Apr 13;11(20):3902–16. doi: 10.1002/cam4.4749 (PMC9582691; doi:10.1002/cam4.4749)

A

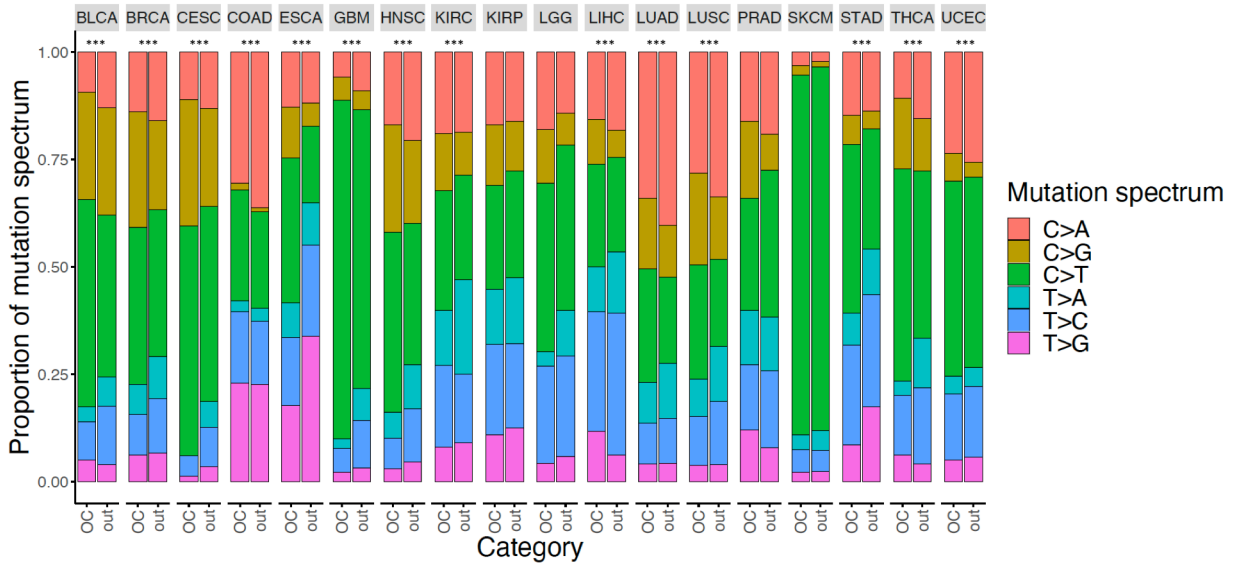

B

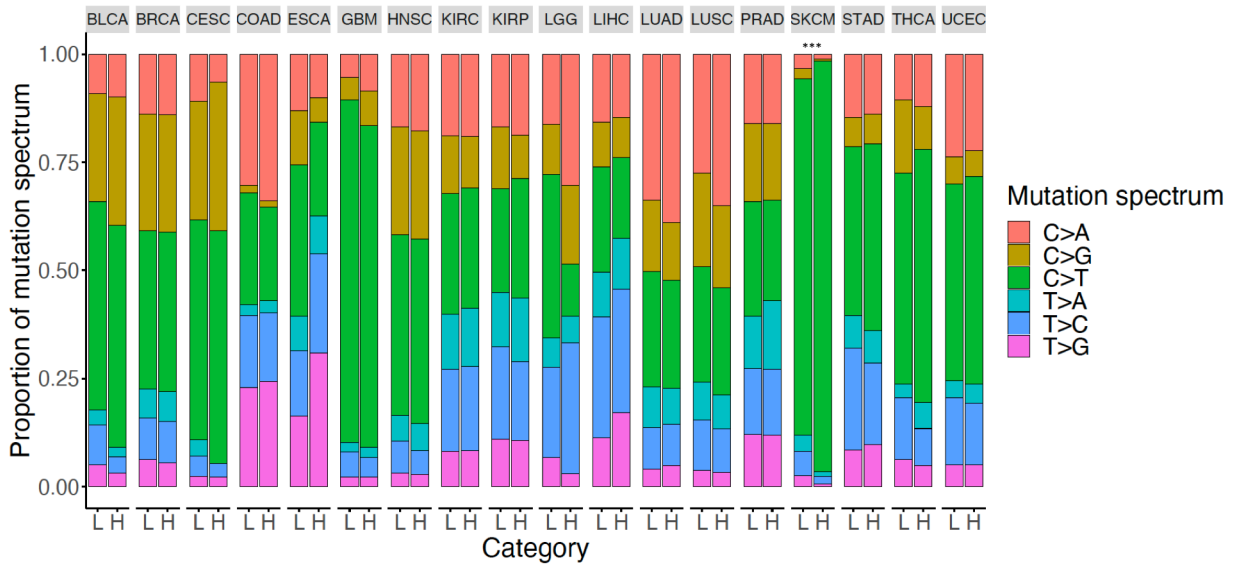

A

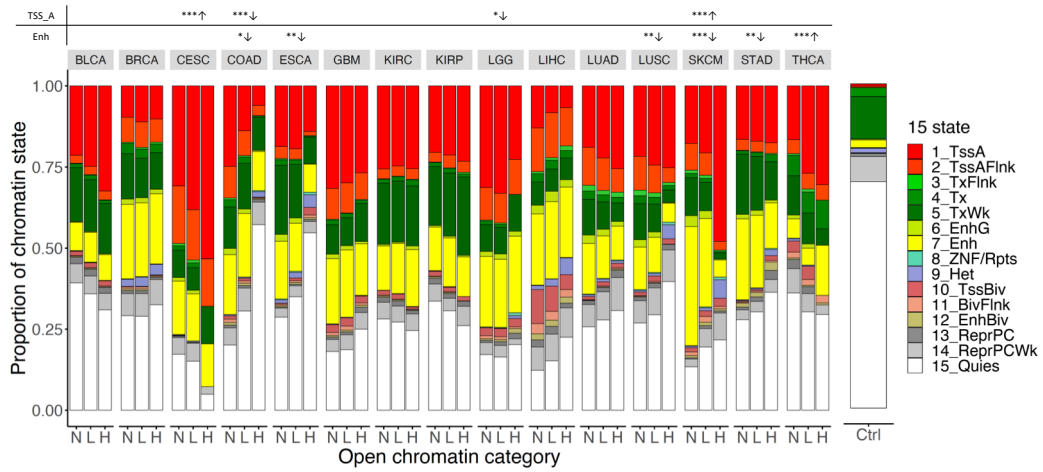

B

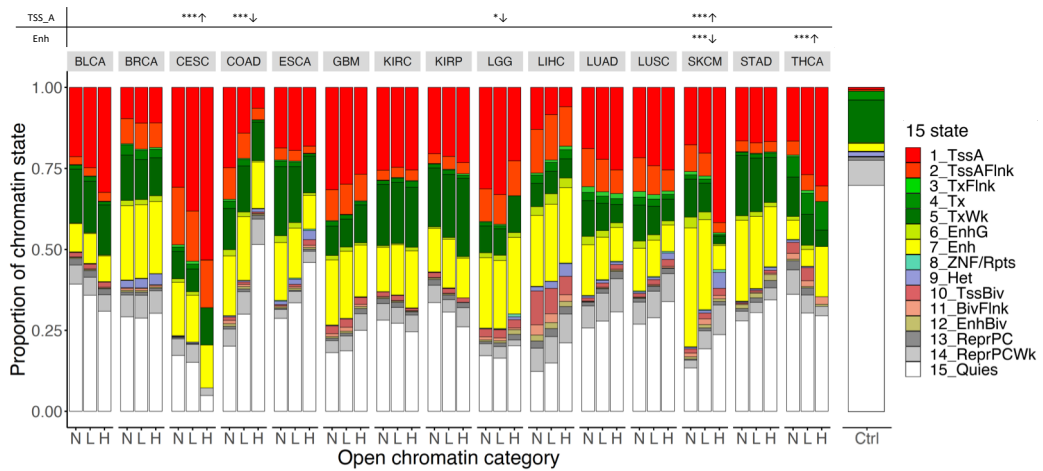

A

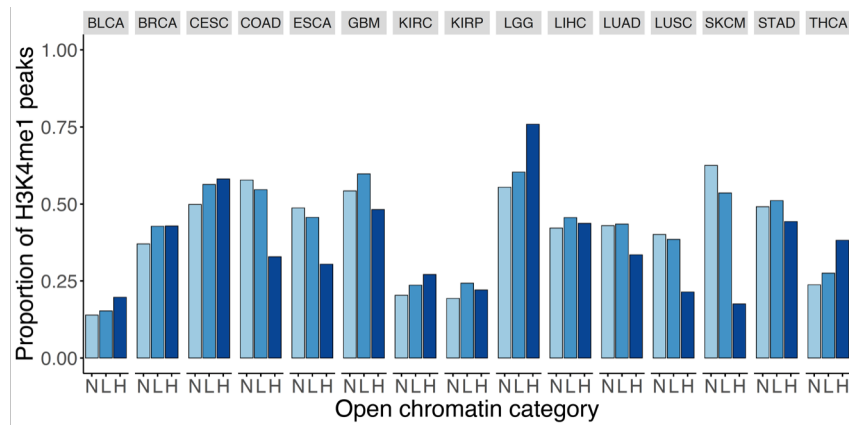

B

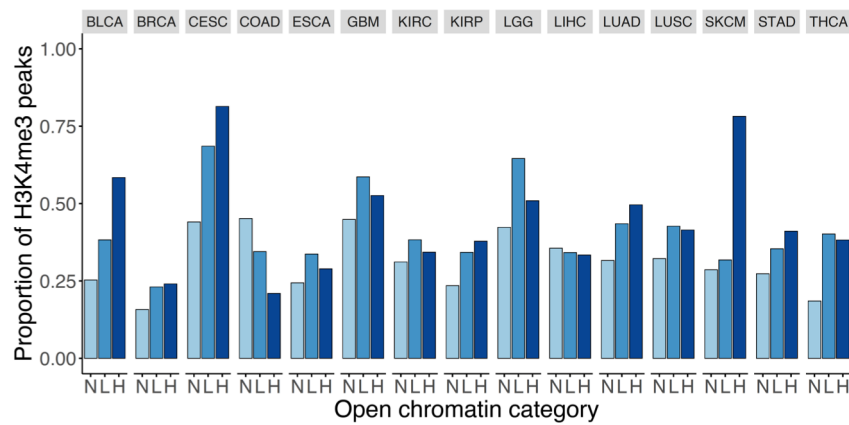

C

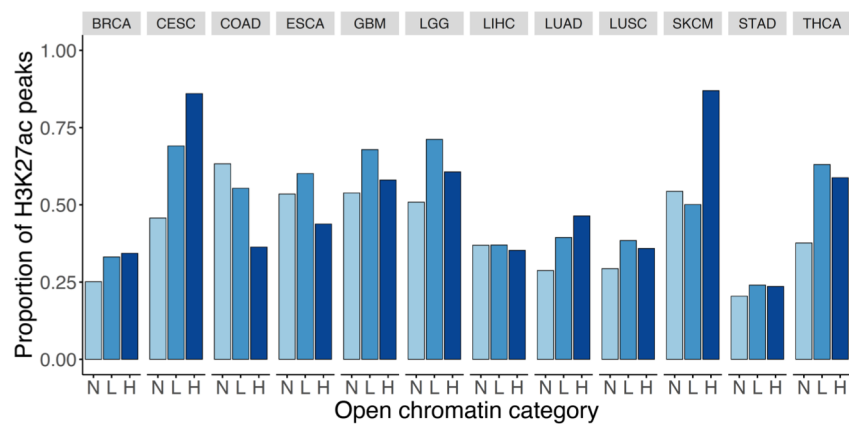

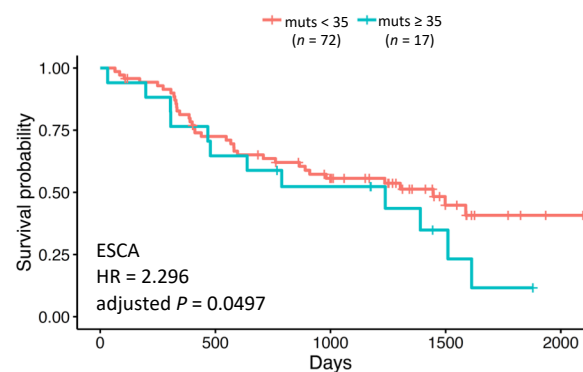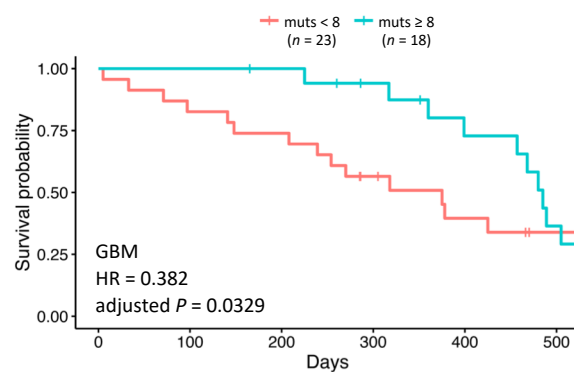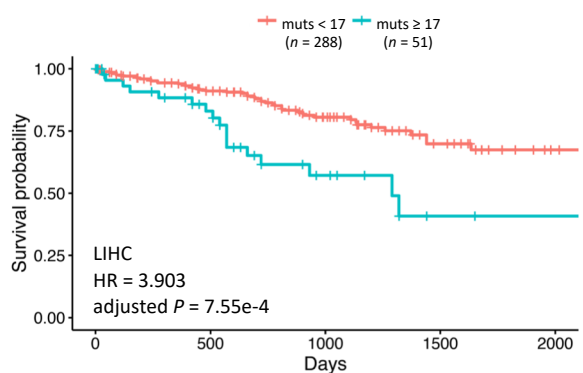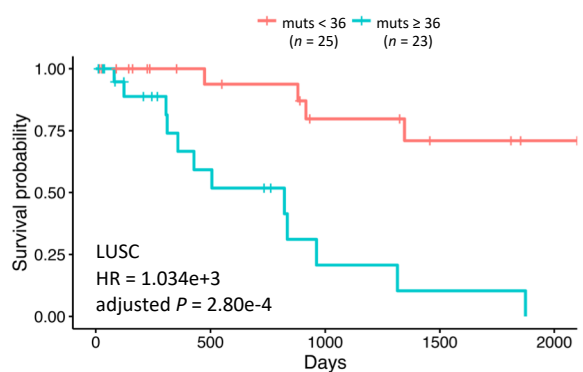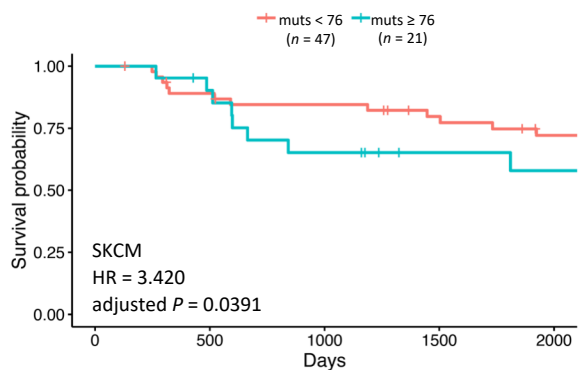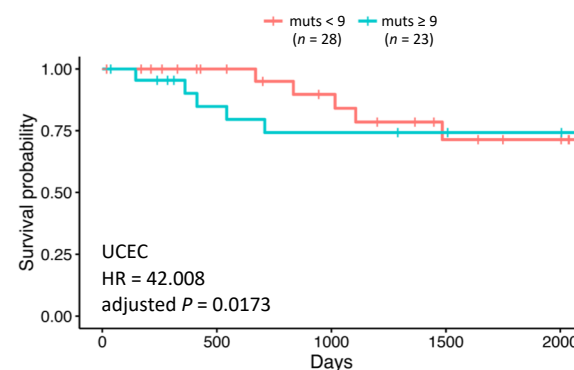

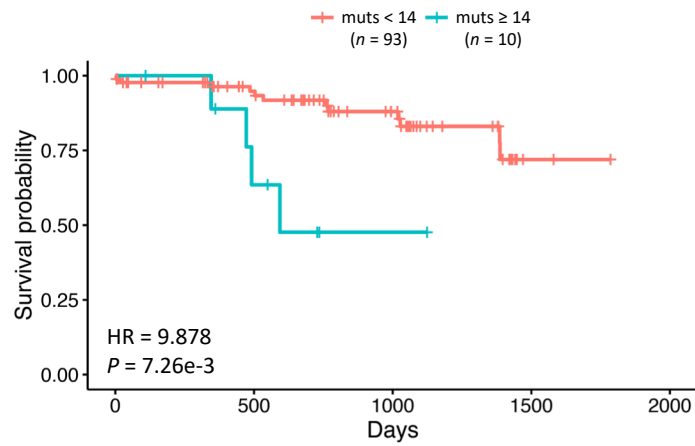

Supplement: Supplementary file 1 — Figures S1‐S5 [file CAM4-11-3902-s001.pdf]
